# Effects of time delays on the therapeutic outcomes of intravenous thrombolysis for acute ischemic stroke in the posterior circulation: An observational study

**DOI:** 10.1002/brb3.1189

**Published:** 2019-01-06

**Authors:** Qiang Huang, Hai‐qing Song, Qing‐feng Ma, Xiao‐wei Song, Jian Wu

**Affiliations:** ^1^ Department of Neurology, Beijing Tsinghua Changgung Hospital, School of Clinical Medicine Tsinghua University Beijing China; ^2^ Department of Neurology, Xuanwu Hospital Capital Medical University Beijing China

**Keywords:** intravenous thrombolysis, outcome, posterior circulation stroke, recanalization, time delay

## Abstract

**Objectives:**

We aim to demonstrate the effects of time delays on the therapeutic outcomes of intravenous thrombolysis (IVT) in acute posterior circulation stroke (PCS) patients.

**Methods:**

Consecutive PCS cases treated with IVT alone were retrospectively examined. The primary end point was set to be a favorable outcome (modified Rankin Scale [mRS] ≤2) at 3 months, and angiographic recanalization was set to be the secondary outcome.

**Results:**

A total of 95 PCS cases with IVT were recruited. The patients with favorable outcomes and those without favorable outcomes had similar baseline characteristics, except for significantly lower National Institute of Health Stroke Scale (NIHSS) scores (5 vs. 12, respectively; *p* < 0.001) and less hyperdense basilar artery signs in head CTs (26.5% vs. 70.4%, respectively; *p* < 0.001) for those with favorable outcomes. For patients with an onset‐to‐treatment time (OTT) of 0–90 min (*n* = 5), 91–180 min (*n* = 38), 181–270 min (*n* = 37), or ≧271 min (*n* = 15), the rate of favorable outcome was 100.0%, 71.1%, 67.6%, or 73.3%, respectively, and the Cochran–Armitage trend test showed no linear trend between the OTT and the clinical prognosis of IVT in PCS (*p* = 0.501) patients. In addition, the rates of recanalization were 100.0%, 68.4%, 64.9%, and 46.7%, and the Cochran–Armitage trend test suggested a linear trend between the OTT and recanalization (*p* = 0.046); that is, the proportion of PCS patients who underwent recanalization decreased with increasing OTTs. In the multivariate logistic regression analysis, after adjusting for confounding factors with *p* ≦ 0.20 in the univariate analysis, baseline NIHSS scores and hyperdense basilar artery signs were negatively associated with favorable outcomes, with odds ratios (OR) of 0.884 (95% confidence interval [CI], 0.804–0.971; *p* = 0.010) and 0.208 (95% CI, 0.062–0.693; *p* = 0.011), respectively. In addition, there was a negative association between recanalization, OTTs (OR, 0.993, 95% CI, 0.987–0.999; *p* = 0.029), and baseline NIHSS scores (OR, 0.881, 95% CI, 0.802–0.967; *p* = 0.008).

**Conclusion:**

Irrespective of stroke severity, the therapeutic effects of recanalization after IVT decreased significantly with longer time delays in PCS patients.

## 1 INTRODUCTION

Although the clinical characteristics between posterior circulation stroke (PCS) and anterior circulation stroke (ACS) are more similar than dissimilar, which makes differentiation between the two using clinical neurological deficits in the absence of the neuroimaging unreliable (Tao et al., 2012), there tends to be different profiles for the response to reperfusion therapy between ACS and PCS patients (Pagola et al., 2011). On the one hand, statistically significant differences were found between PCS and ACS patients treated with intravenous thrombolysis (IVT), including more favorable outcomes (Sarikaya et al., 2011) and a reduced risk of intracranial hemorrhage (ICH) in PCS patients (Dornak et al., 2015; Sarikaya et al., 2011). Moreover, compared with ACS patients, PCS patients also experienced longer time delays before receiving a neurology evaluation after the initial emergency department evaluation and longer delays before the administration of IVT (Sarraj et al., 2015). It is worth noting, however, that differences between PCS and ACS in regard to the efficacy and safety of IVT have not been previously investigated using randomized controlled trials (RCTs; Hacke et al., 2008; the NINDS trial., 1995). On the other hand, although endovascular therapy with stent retrievers has been shown to have increased efficacy compared with IVT in several RCTs (Sardar et al., 2015), as mentioned in the American Heart Association/American Stroke Association guidelines, the analyzed studies included so few PCS patients (8%) that the superiority of endovascular therapy over IVT for PCS is still uncertain (Powers et al., 2015). Therefore, although earlier treatment has been strongly associated with increased proportional IVT benefits for all ischemic stroke patients (Emberson et al., 2014), the effects of time delays on the therapeutic outcomes of IVT in PCS patients must be further evaluated.

## 2 METHODS

A standard stroke pathway was available for 7 days a week and 24 hr a day in two tertiary teaching hospitals, which were described in detail in our previous study (Huang et al., 2016). Consecutive AIS patients treated with IVT in these two hospitals were collected by the same team from March 2011 to December 2017 and were enrolled in this analysis. We retrospectively identified consecutive PCS cases treated with IVT from the two databases. The detailed inclusion process for IVT cases was mentioned in previous studies (Huang et al., 2016, 2015). The primary inclusions and exclusions for IVT followed published Chinese guidelines (Liu et al., 2010). Briefly, the following guidelines were used: (a) a definite and persistent neurological deficit (as measured by National Institute of Health Stroke Scale (NIHSS) score), (b) age ≥18 years, (c) onset‐to‐treatment time (OTT) ≤4.5 hr, (d) no intracranial hemorrhage (ICH) in the screening head CT and no other known contraindications of IVT therapy, and (e) written informed consents were received from the patients or their proxies. Recombinant tissue‐type plasminogen activator (rt‐PA), at a standard dose of 0.9 mg/kg (maximum 90 mg), was using as the thrombolytic agent, which was applied as recommended in the guidelines (Liu et al., 2010). Routine head imaging (CT or MR) was performed within 24 hr to identify responsible cerebral infarction lesions and to exclude intracranial hemorrhagic complications before the initiation of treatment with antiplatelet agents. Transcranial Doppler (TCD) and ultrasound for the cervical arteries were performed within 24 hr, and vascular imaging (CT or MR angiography) was performed within 36 hr to detect vascular lesions. Lesion sites (classified as anterior or posterior circulation) and their responsible cerebral vascular territories (classified as vertebral artery, basilar artery, and posterior cerebral artery) were confirmed according to the results of follow‐up head CT or MR imaging. The baseline characteristics, the results of screening tests, and the outcome measures were collected on case report forms by experienced stroke team members. Follow‐up was conducted through a structured telephone interview at 3 months.

### 2.1 Explanatory and outcome variables

All PCS patient information was collected on a standard case report form. Demographic data (sex and age), medical history (hypertension, diabetes, dyslipidemia, coronary heart disease, atrial fibrillation, and prior stroke), smoking and drinking status, baseline variables (body mass index, OTT, blood pressure, blood glucose, NIHSS, and hyperdense basilar artery [HDBA] signs in the head CT), and outcome measures (modified Rankin Scale [mRS], recanalization status, ICH, and symptomatic intracranial hemorrhage [sICH]) were included in the analysis. Stroke onset was defined as the time when definite neurological symptoms first occurred or the last time known to be normal, and the treatment time was defined as the time of administration of the bolus dose of rt‐PA for IVT. OTT was further divided into 0–90, 91–180, 181–270, and ≧271 min subgroups.

The mRS score (with a cutoff ≤2 as a favorable outcome) at 3 month and vascular recanalization were taken as the primary and secondary therapeutic end points, respectively. Recanalization of the responsible cerebral vessel after IVT was first detected by a 24‐hr TCD with a preset criteria (e.g., a moderate to severe degree of stenosis in the basilar artery was defined as ≥50%, which was assessed as a peak systolic velocity ≥100 cm/s and featured spectrum or audio frequency; Hua, Gao, Wu, Pan, & Qian, 2008) and further confirmed by CT or MR angiography. Other outcomes included sICH (defined as any decline in the NIHSS score accompanied by a hemorrhage observed in the head CT imaging within 36 hr after the treatment) and mortality at 3 months.

### 2.2 Ethics approval and consent to participate

The study protocol and data analysis were approved by the Ethical Committee of Beijing Tsinghua Changgung Hospital and by the Ethical Committee of Xuanwu Hospital, and conform to the principles outlined in the Declaration of Helsinki. Written informed consent for IVT was obtained from all patients.

### 2.3 Statistical analysis

SPSS 19.0 software was used for statistical calculations, with a two‐tailed *p < *0.05 defined as being statistically significant. A Mann–Whitney *U* test and a Pearson chi‐squared test were used for group comparisons of related variables. A Cochran–Armitage trend test was conducted to detect the potential linear trend between OTT subgroups and therapeutic outcomes. Multivariate logistic regression analysis, adjusted by confounding factors with *p ≤ *0.20 in the univariate analysis, was performed to identify the predictors of favorable outcome and recanalization. Effect sizes were presented as odds ratios (ORs), and statistical uncertainty was presented as the 95% confidence interval (CI).

## 3 RESULTS

A total of 95 PCS cases with IVT therapy (69 cases were enrolled between March 2011 and December 2015 in Beijing Xuanwu hospital and 26 cases enrolled between January 2016 and December 2017 in Beijing Tsinghua Changgung Hospital) were identified. Basic characteristics for the total group and the PCS subgroups are outlined in Tables 1 and 2. For all recruited cases, the median age was 64 (55, 73) years, 30.5% were female, the median NIHSS score was 6 (4, 11), and the median OTT was 199 (140, 245) min. The rates of lesion site in responsible cerebral arteries for vertebral artery, basilar artery, and posterior cerebral artery were 13.7%, 48.4%, and 37.9%, respectively. The patients with favorable outcomes (of both a mRS score ≦2 at 3 months and recanalization) and those without favorable outcomes had similar baseline characteristics, except for significantly lower National Institute of Health Stroke Scale (NIHSS) scores, less hyperdense basilar artery signs in head CTs, and occlusive basilar arteries in the favorable outcome group. The rates of favorable outcomes (mRS ≦2 at 3 months), recanalization, sICH, and mortality in the total population were 71.6% (68/95), 65.3% (62/95), 4.2% (4/95), and 8.4% (8/95), respectively. There were much lower rates of sICH (0% vs. 14.8%; *p* = 0.001) and mortality (0% vs. 29.6%; *p* < 0.001) in the patients with favorable outcomes than in those without favorable outcomes.

**Table 1:** Baseline characteristics of included cases

	Total population (*n* = 95)	Subgroups			Subgroups		
		mRS≦2 (*n* = 68)	mRS＞2 (*n* = 27)	*p*	Recanalization (*n* = 62)	Nonrecanalization (*n* = 33)	*p*
Age (years)	64 (55–73)	66 (55–73)	63 (51–71)	0.418	65 (55–73)	63 (53–72)	0.693
Female	29 (30.5)	17 (25.0)	12 (44.4)	0.063	17 (27.4)	12 (36.4)	0.367
Medical history							
Hypertension	60 (63.2)	41 (60.3)	19 (70.4)	0.358	40 (64.5)	20 (60.6)	0.707
Diabetes	36 (37.9)	27 (39.7)	9 (33.3)	0.564	23 (37.1)	13 (39.4)	0.826
Dyslipidemia	39 (41.1)	30 (44.1)	9 (33.3)	0.335	27 (43.5)	12 (36.4)	0.498
Coronary heart disease	15 (15.8)	9 (13.2)	6 (22.2)	0.279	9 (14.5)	6 (18.2)	0.641
Atrial fibrillation	8 (8.4)	4 (5.9)	4 (14.8)	0.157	3 (4.9)	5 (15.2)	0.085
Prior stroke	22 (23.2)	13 (19.1)	9 (33.3)	0.138	14 (22.6)	8 (24.2)	0.855
Current smoking	41 (43.2)	31 (45.6)	10 (37.0)	0.448	27 (43.5)	14 (42.4)	0.916
Heavy drinking	25 (26.3)	17 (25.0)	8 (29.6)	0.644	16 (25.8)	9 (27.3)	0.877
Onset‐to‐treatment time	199 (140–245)	194 (136–237)	206 (150–252)	0.198	186 (126–232)	206 (170–266)	0.030
Baseline NIHSS score	6 (4–11)	5 (3–8)	12 (8–16)	<0.001	5 (3–8)	10 (5–16)	<0.001
Baseline SBP (mmHg)	154 (140–170)	153 (140–170)	155 (140–165)	0.682	151 (135–170)	157 (143–170)	0.655
Baseline DBP (mmHg)	84 (75–90)	83 (71–90)	80 (73–93)	0.622	84 (77–90)	85 (73–96)	0.966
Blood sugar (mmol/L)	7.9 (6.0–10.9)	7.7 (6.0–10.6)	8.8 (5.9–12.0)	0.460	7.4 (6.0–10.5)	8.7 (7.0–12.1)	0.098
Hyperdense basilar artery signs	37 (38.9)	18 (26.5)	19 (70.4)	<0.001	18 (29.0)	19 (57.6)	0.007
Responsible occlusive cerebral artery							
Vertebral artery	13 (13.7)	10 (14.7)	3 (11.1)	0.022	7 (11.3)	6 (18.2)	0.015
Basilar artery	46 (48.4)	27 (39.7)	19 (70.4)		25 (40.3)	21 (63.6)	
Posterior cerebral artery	36 (37.9)	31 (45.6)	5 (18.5)		30 (48.4)	6 (18.2)	

Notes

If not otherwise stated, continuous data are presented as the median (IQR).

DBP, diastolic blood pressure; IQR, interquartile range; mRS, modified Rankin Scale; NIHSS, National Institutes of Health Stroke Scale; SBP, systolic blood pressure.

**Table 2:** Baseline characteristics of included cases in different OTT subgroups

	Subgroup with OTT 0–90 min (*n* = 5)	Subgroup with OTT 91–180 min (*n* = 38)	Subgroup with OTT 181–270 min (*n* = 37)	Subgroup with OTT ≧271 min (*n* = 15)	*p*
Age (years)	69 (67–75)	65 (55–72)	62 (54–73)	66 (51–69)	0.451
Female	1 (20.0)	8 (21.1)	13 (35.1)	7 (46.7)	0.253
Medical history					
Hypertension	2 (40.0)	24 (63.2)	22 (59.5)	12 (80.0)	0.362
Diabetes	4 (80.0)	14 (36.7)	10 (27.0)	8 (53.3)	0.067
Dyslipidemia	1 (20.0)	16 (42.1)	17 (45.9)	5 (33.3)	0.644
Coronary heart disease	1 (20.0)	7 (18.4)	5 (13.5)	2 (13.3)	0.924
Atrial fibrillation	1 (20.0)	1 (2.6)	5 (13.5)	1 (6.7)	0.281
Prior stroke	0 (0)	11 (28.9)	8 (21.6)	3 (20.0)	0.502
Current smoking	3 (60.0)	16 (42.1)	14 (37.8)	8 (53.3)	0.647
Heavy drinking	0 (0)	10 (26.3)	11 (29.8)	4 (26.6)	0.571
Baseline NIHSS score	13 (4–18)	5 (4–12)	8 (4–10)	6 (40.0)	0.344
Baseline SBP (mmHg)	140 (120–157)	158 (140–170)	154 (132–167)	150 (145–170)	0.403
Baseline DBP (mmHg)	75 (69–90)	86 (75–90)	85 (79–96)	84 (73–90)	0.770
Blood sugar (mmol/L)	17.5 (8.7–18.7)	6.8 (5.8–8.9)	8.0 (5.9–11.2)	10.5 (7.4–15.9)	0.007
Hyperdense basilar artery signs	2 (40.0)	17 (44.7)	13 (35.1)	5 (33.3)	0.810
Responsible occlusive cerebral artery					
Vertebral artery	0 (0)	6 (15.8)	4 (10.8)	3 (20.0)	0.039
Basilar artery	0 (0)	20 (52.6)	17 (45.9)	9 (60.0)	
Posterior cerebral artery	5 (100.0)	12 (31.6)	16 (43.2)	3 (20.0)	

Notes

If not otherwise stated, continuous data are presented as median (IQR).

DBP, diastolic blood pressure; IQR, interquartile range; mRS, modified Rankin Scale; NIHSS, National Institutes of Health Stroke Scale; OTT, onset‐to‐treatment time; SBP, systolic blood pressure.

All the baseline characteristics, except for blood sugar (*p* = 0.007) and responsible occlusive cerebral artery (*p* = 0.039), had similar distributions among the various OTT subgroups (Table 2). The rates of favorable outcomes in patients with OTTs of 0–90 min (*n* = 5), 91–180 min (*n* = 38), 181–270 min (*n* = 37), and ≧271 min (*n* = 15) were 100.0%, 71.1%, 67.6%, and 73.3%, respectively, and the results of the Cochran–Armitage trend test showed that there was no linear trend between the OTT and the clinical prognosis of IVT in PCS patients (*p* = 0.501). In addition, the rates of recanalization in patients with OTTs of 0–90 min (*n* = 5), 91–180 min (*n* = 38), 181–270 min (*n* = 37), and ≧271 min (*n* = 15) were 100.0%, 68.4%, 64.9%, and 46.7%, respectively. The results of the Cochran–Armitage trend test suggested that there was a linear trend between OTT and recanalization after IVT in PCS patients (*p* = 0.046); that is, the proportion of patients who underwent recanalization decreased with increasing OTTs in PCS patients.

In the multivariate logistic regression analysis, after adjusting for confounding factors with *p *≦ 0.20 in the univariate analysis (Tables 3 and 4), baseline NIHSS scores and hyperdense basilar artery signs were negatively associated with favorable outcomes, with odds ratios (OR) of 0.884 (95% confidence interval [CI], 0.804–0.971; *p* = 0.010) and 0.208 (95% CI, 0.062–0.693; *p* = 0.011), respectively. In addition, there were negative associations between recanalization, OTT (OR, 0.993, 95% CI, 0.987–0.999; *p* = 0.029), and baseline NIHSS scores (OR, 0.881, 95% CI, 0.802–0.967; *p* = 0.008).

**Table 3:** Factors associated with mRS ≦ 2 at 3 months in the multivariate logistic regression models (*n* = 95)

	OR	95% CI	*p *Value
Sex	1.447	0.443–4.729	0.541
Atrial fibrillation	0.553	0.080–3.811	0.547
Prior stroke	0.305	0.089–1.045	0.059
Onset‐to‐needle time	0.995	0.988–1.002	0.169
Baseline NIHSS score	0.884	0.804–0.971	0.010
Hyperdense basilar artery signs	0.208	0.062–0.693	0.011

Notes

Other variables (with *p *˃ 0.20 in the univariate analysis) were not included in the multivariate analysis.

CI, confidence interval; NIHSS, National Institutes of Health Stroke Scale; OR, odds ratio.

**Table 4:** Factors associated with recanalization in the multivariate logistic regression models (*n* = 95)

	OR	95% CI	*p *Value
Atrial fibrillation	0.179	0.053–1.727	0.303
Onset‐to‐needle time	0.993	0.987–0.999	0.029
Baseline NIHSS score	0.881	0.802–0.967	0.008
Hyperdense basilar artery signs	0.490	0.168–1.430	0.192
Blood sugar	0.942	0.840–1.057	0.310

Notes

Other variables (with *p *˃ 0.20 in the univariate analysis) were not included in the multivariate analysis.

CI, confidence interval; mRS, modified Rankin Scale; NIHSS, National Institutes of Health Stroke Scale; OR, odds ratio.

## 4 DISCUSSION

Our results showed that, irrespective of stroke severity, a linear trend existed between OTT and recanalization (*p* = 0.046 in the Cochran–Armitage trend test), with an OR of 0.993 per minute (95% CI, 0.987–0.999; *p* = 0.029), but not between OTT and favorable outcomes (*p* = 0.501 in the Cochran–Armitage trend test) in PCS patients treated with IVT. That is, despite a greater ischemic tolerance (Pagola et al., 2007) and a safer profile in bleeding complications (Sarikaya et al., 2011) when compared with ACS patients, recanalization after IVT was still time‐dependent for PCS patients, which was consistent with the results reported for ACS patients.

Given to the fact that PCS cases, especially those with basilar artery occlusion (BAO), may naturally follow a dismal course (Schonewille, Algra, Serena, Molina, & Kappelle, 2005), timely recanalization may represent the only solution (Kumar, Shahripour, & Alexandrov, 2014). As shown in our study, the differences between PCS and ACS in regard to ischemic tolerance and the response to hemorrhagic complication do not appear to change the time‐dependent nature of vascular recanalization. This result was also supported by the strong association between AIS treated with IVT within the first hour (the golden hour) and the substantially higher chance of complete recanalization and the early reversal of neurological deficits reported in the results of the Safe Implementation of Treatments in Stroke–East registry (Tsivgoulis et al., 2017). Moreover, a strong association between late recanalization (˃6 hr) and unfavorable outcomes for BAO patients after endovascular therapy was also observed in the Basilar Artery International Cooperation Study (BASICS; Vergouwen et al., 2012). These results may be due to the benefits of a higher rate of recanalization after endovascular therapy being offset by longer time delays in OTT. Therefore, efforts made to reduce time delays for IVT and to increase the rate of patients treated in the “golden hour,” such as with the application of treatment in novel settings with a mobile stroke unit and the optimization of work flows in traditional hospital settings, deserve increased attention and should be generalized (Huang, Zhang, Xu, & Wu, 2018). It is noteworthy that there were no significant differences in the safety and efficacy of ultra‐early IVT between the two settings (Tsivgoulis, Geisler, et al., 2018; Tsivgoulis, Katsanos, et al., 2018), highlighting the key role of prompt IVT initiation, regardless of treatment location.

High morbidity (28.4%) and mortality (8.4%) were observed in PCS patients in our study, even after standard IVT, which was consistent with the results of previous studies (Kumar et al., 2014; Lindsberg et al., 2004). However, the optimal interventions for PCS are still uncertain (Powers et al., 2015) and could include IVT, IVT bridging therapy, or advanced endovascular therapy (with stent retrievers (Mordasini et al., 2013) or endovascular sonolysis (Kuliha et al., 2013)). A recent meta‐analysis showed that pretreatment with IVT in patients with large vessel occlusions only resulted in a successful recanalization rate of approximately 10%, with patients requiring additional endovascular therapy (Tsivgoulis, Geisler, et al., 2018; Tsivgoulis, Katsanos, et al., 2018). The advanced endovascular therapy with the highest rate of recanalization is the most likely to become the best treatment for PCS, according to the similar history of reperfusion therapy for acute myocardial infarction (Widimsky, Coram, & Abou‐Chebl, 2014) and for ACS (Sardar et al., 2015; Tsivgoulis, Geisler, et al., 2018; Tsivgoulis, Katsanos, et al., 2018). Inspired by the rapid progress being made in endovascular therapy techniques, the overall recanalization rate of BAO patients using novel stent retrievers (Mordasini et al., 2013) or endovascular sonolysis (Kuliha et al., 2013) could reach as high as 100%, and the rate of ICH has been relatively low, which demonstrates the greatly improved benefits and reduced complications of endovascular therapy. However, it is still worth mentioning that a streamlined organization with reduced time delays in stroke care plays a key role in delivering the benefits of reperfusion therapies for both PCS and ACS patients.

The blood clot load can contribute to another important factor in revascularization for PCS cases. HDBA signs in the CT scan might serve as a valuable marker of PCS (especially for BAO cases). As shown in a previous study (Goldmakher et al., 2009), HDBA signs can be utilized to detect thrombosis in the basilar artery and to predict poor outcomes in patients presenting PCS symptoms. HDBA signs were also a negative predictor for favorable outcomes of PCS cases in our study, which was consistent with the results of a previous study (Goldmakher et al., 2009). However, the low incidence (38.9% in our study) of HDBA signs in the admission CT could restrict its utility, and the accuracy must be further validated.

The limitations of this study primarily result from the small sample size and the retrospective study design. Moreover, severe PCS cases were more likely to receive endovascular therapy, which could have induced a selective bias. In addition, the clinical outcome follow‐up being conducted through telephone interviews could have resulted in the subjective bias of the patients. Finally, our study has the same limits faced by all non‐RCTs.

## 5 CONCLUSION

Our study demonstrated a significant association and linear trend between OTT and vascular recanalization in PCS cases after IVT, in which the concept of “time is brain” was further highlighted for PCS, with greater ischemic tolerance than ACS.

## CONFLICT OF INTEREST

The authors declare that they have no competing interests.

## AUTHOR CONTRIBUTIONS

Qiang Huang, Xiao‐wei Song, Qing‐feng Ma, Hai‐qing Song, contributed to data curation. Qiang Huang, wrote the original draft. Qiang Huang, contributed to methodology. Jian Wu, contributed to writing—review and editing. Qiang Huang, performed formal analysis. Jian, Wu involved in project administration.

## References

[brb31189-bib-0001] Dornak, T. , Kral, M. , Hazlinger, M. , Herzig, R. , Veverka, T. , Burval, S. , … Kanovsky, P. (2015). Posterior vs. anterior circulation infarction: Demography, outcomes, and frequency of hemorrhage after thrombolysis. International Journal of Stroke, 10, 1224–1228. 10.1111/ijs.12626 26310390

[brb31189-bib-0002] Emberson, J. , Lees, K. R. , Lyden, P. , Blackwell, L. , Albers, G. , Bluhmki, E. , … Hacke, W. (2014). Effect of treatment delay, age, and stroke severity on the effects of intravenous thrombolysis with alteplase for acute ischaemic stroke: A meta‐analysis of individual patient data from randomised trials. Lancet, 384, 1929–1935. 10.1016/S0140-6736(14)60584-5 25106063 PMC4441266

[brb31189-bib-0003] Goldmakher, G. V. , Camargo, E. C. , Furie, K. L. , Singhal, A. B. , Roccatagliata, L. , Halpern, E. F. , … Lev, M. H. (2009). Hyperdense basilar artery sign on unenhanced CT predicts thrombus and outcome in acute posterior circulation stroke. Stroke, 40, 134–139. 10.1161/STROKEAHA.108.516690 19038918

[brb31189-bib-0004] Hacke, W. , Kaste, M. , Bluhmki, E. , Brozman, M. , Davalos, A. , Guidetti, D. , … Toni, D. (2008). Thrombolysis with alteplase 3 to 4.5 hours after acute ischemic stroke. New England Journal of Medicine, 359, 1317–1329. 10.1056/NEJMoa0804656 18815396

[brb31189-bib-0005] Hua, Y. , Gao, S. , Wu, G. , Pan, X. , & Qian, S. (2008). A guide for operation norm and diagnostic criteria of transcranial Doppler. 197–222.

[brb31189-bib-0006] Huang, Q. , Song, H. Q. , Ji, X. M. , Cheng, W. Y. , Feng, J. , Wu, J. , & Ma, Q. F. (2016). Generalization of the right acute stroke prevention strategies in reducing in‐hospital delays. PLoS ONE, 11, e154972 10.1371/journal.pone.0154972 PMC4859531 27152854

[brb31189-bib-0007] Huang, Q. , Zhang, J. Z. , Xu, W. D. , & Wu, J. (2018). Generalization of the right acute stroke promotive strategies in reducing delays of intravenous thrombolysis for acute ischemic stroke: A meta‐analysis. Medicine (Baltimore), 97, e11205 10.1097/MD.0000000000011205 29924046 PMC6024468

[brb31189-bib-0008] Huang, Q. , Ma, Q. F. , Feng, J. , Cheng, W. Y. , Jia, J. P. , Song, H. Q. , … Wu, J. (2015). Factors associated with in‐hospital delay in intravenous thrombolysis for acute ischemic stroke: Lessons from China. PLoS ONE, 10, e143145 10.1371/journal.pone.0143145 PMC4648585 26575839

[brb31189-bib-0009] Kuliha, M. , Roubec, M. , Jonszta, T. , Krajca, J. , Czerny, D. , Krajina, A. , … Skoloudik, D. (2013). Safety and efficacy of endovascular sonolysis using the EkoSonic endovascular system in patients with acute stroke. American Journal of Neuroradiology, 34, 1401–1406. 10.3174/ajnr.A3416 23370469 PMC8051501

[brb31189-bib-0010] Kumar, G. , & Shahripour, R. B. , & Alexandrov, A. V. (2014). Recanalization of acute basilar artery occlusion improves outcomes: A meta‐analysis. Journal of NeuroInterventional Surgery, 7(12), 868–874. 10.1136/neurintsurg-2014-011418 25271064

[brb31189-bib-0011] Lindsberg, P. J. , Soinne, L. , Tatlisumak, T. , Roine, R. O. , Kallela, M. , Happola, O. , & Kaste, M. (2004). Long‐term outcome after intravenous thrombolysis of basilar artery occlusion. JAMA, 292, 1862–1866. 10.1001/jama.292.15.1862 15494584

[brb31189-bib-0012] Liu, M. , Zhang, S‐M. , Rao, M.‐L. , Lv, C.‐Z. , Wang, J.‐Z. , Huang, R.‐X. , & Dong, Q. (2010). Chinese guidelines for diagnosis and management of acute ischemic stroke 2010. pp. 146–153.

[brb31189-bib-0013] Mordasini, P. , Brekenfeld, C. , Byrne, J. V. , Fischer, U. , Arnold, M. , Heldner, M. R. , … Gralla, J. (2013). Technical feasibility and application of mechanical thrombectomy with the Solitaire FR Revascularization device in acute basilar artery occlusion. American Journal of Neuroradiology, 34, 159–163. 10.3174/ajnr.A3168 22723058 PMC7966341

[brb31189-bib-0014] National Institute of Neurological Disorders and Stroke rt‐PA Stroke Study Group (the NINDS trial) . (1995). Tissue plasminogen activator for acute ischemic stroke. New England Journal of Medicine, 333, 1581–1587. 7477192 10.1056/NEJM199512143332401

[brb31189-bib-0015] Pagola, J. , Ribo, M. , Alvarez‐Sabin, J. , Lange, M. , Rubiera, M. , & Molina, C. A. (2007). Timing of recanalization after microbubble‐enhanced intravenous thrombolysis in basilar artery occlusion. Stroke, 38, 2931–2934. 10.1161/STROKEAHA.107.487454 17901389

[brb31189-bib-0016] Pagola, J. , Ribo, M. , Alvarez‐Sabin, J. , Rubiera, M. , Santamarina, E. , Maisterra, O. , … Molina, C. A. (2011). Thrombolysis in anterior versus posterior circulation strokes: Timing of recanalization, ischemic tolerance, and other differences. Journal of Neuroimaging, 21, 108–112. 10.1111/j.1552-6569.2009.00462.x 20040010

[brb31189-bib-0017] Powers, W. J. , Derdeyn, C. P. , Biller, J. , Coffey, C. S. , Hoh, B. L. , Jauch, E. C. , … Yavagal, D. R. (2015). 2015 American Heart Association/American Stroke Association Focused Update of the 2013 Guidelines for the early management of patients with acute ischemic stroke regarding endovascular treatment: A guideline for healthcare professionals from the American Heart Association/American Stroke Association. Stroke, 46, 3020–3035. 10.1161/STR.0000000000000074 26123479

[brb31189-bib-0018] Sardar, P. , Chatterjee, S. , Giri, J. , Kundu, A. , Tandar, A. , Sen, P. , … Gray, W. A. (2015). Endovascular therapy for acute ischaemic stroke: A systematic review and meta‐analysis of randomized trials. European Heart Journal, 36, 2373–2380. 10.1093/eurheartj/ehv270 26071599

[brb31189-bib-0019] Sarikaya, H. , Arnold, M. , Engelter, S. T. , Lyrer, P. A. , Mattle, H. P. , Georgiadis, D. , … Baumgartner, R. W. (2011). Outcomes of intravenous thrombolysis in posterior versus anterior circulation stroke. Stroke, 42, 2498–2502. 10.1161/STROKEAHA.110.607614 21778443

[brb31189-bib-0020] Sarraj, A. , Medrek, S. , Albright, K. , Martin‐Schild, S. , Bibars, W. , Vahidy, F. , … Savitz, S. I. (2015). Posterior circulation stroke is associated with prolonged door‐to‐needle time. International Journal of Stroke, 10, 672–678. 10.1111/j.1747-4949.2012.00952.x 23521891

[brb31189-bib-0021] Schonewille, W. J. , Algra, A. , Serena, J. , Molina, C. A. , & Kappelle, L. J. (2005). Outcome in patients with basilar artery occlusion treated conventionally. Journal of Neurology, Neurosurgery and Psychiatry, 76, 1238–1241. 10.1136/jnnp.2004.049924 PMC1739786 16107358

[brb31189-bib-0022] Tao, W. D. , Liu, M. , Fisher, M. , Wang, D. R. , Li, J. , Furie, K. L. , … Wu, B. (2012). Posterior versus anterior circulation infarction: How different are the neurological deficits? Stroke, 43, 2060–2065. 10.1161/STROKEAHA.112.652420 22678088

[brb31189-bib-0023] Tsivgoulis, G. , Geisler, F. , Katsanos, A. H. , Korv, J. , Kunz, A. , Mikulik, R. , … Audebert, H. J. (2018). Ultraearly intravenous thrombolysis for acute ischemic stroke in mobile stroke unit and hospital settings. Stroke, 49, 1996–1999. 10.1161/STROKEAHA.118.021536 29986934

[brb31189-bib-0024] Tsivgoulis, G. , Katsanos, A. H. , Kadlecova, P. , Czlonkowska, A. , Kobayashi, A. , Brozman, M. , … Mikulik, R. (2017). Intravenous thrombolysis for ischemic stroke in the golden hour: Propensity‐matched analysis from the SITS‐EAST registry. Journal of Neurology, 264, 912–920. 10.1007/s00415-017-8461-8 28315960

[brb31189-bib-0025] Tsivgoulis, G. , Katsanos, A. H. , Schellinger, P. D. , Kohrmann, M. , Varelas, P. , Magoufis, G. , … Alexandrov, A. V. (2018). Successful reperfusion with intravenous thrombolysis preceding mechanical thrombectomy in large‐vessel occlusions. Stroke, 49, 232–235. 29212743 10.1161/STROKEAHA.117.019261 PMC5742056

[brb31189-bib-0026] Vergouwen, M. D. , Algra, A. , Pfefferkorn, T. , Weimar, C. , Rueckert, C. M. , Thijs, V. , … Schonewille, W. J. (2012). Time is brain(stem) in basilar artery occlusion. Stroke, 43, 3003–3006. 22989501 10.1161/STROKEAHA.112.666867

[brb31189-bib-0027] Widimsky, P. , Coram, R. , & Abou‐Chebl, A. (2014). Reperfusion therapy of acute ischaemic stroke and acute myocardial infarction: Similarities and differences. European Heart Journal, 35, 147–155. 10.1093/eurheartj/eht409 24096325 PMC3890694

